# Effect of Biological Therapy on Symptom Burden and Functional Disability in Patients with Migraine: A Before-and-After Analysis

**DOI:** 10.2478/sjph-2026-0005

**Published:** 2026-03-01

**Authors:** Albina Ličina, Andrej Fabjan, Janez Žibert, Tina Kamenšek

**Affiliations:** University Medical Centre Ljubljana, Department of Neurology, Zaloška cesta 2a, 1000 Ljubljana, Slovenia; University of Ljubljana, Faculty of Health Sciences, Zdravstvena pot 5, 1000 Ljubljana, Slovenia

**Keywords:** Monoclonal antibodies, Neurology, Disability evaluation, Preventive treatment, monoklonska protitelesa, nevrologija, ocenjevanje invalidnosti, preventivno zdravljenje

## Abstract

**Introduction:**

This study aims to assess the effectiveness of biological therapies in reducing migraine-related symptom burden and functional disability, including reductions in monthly migraine days, pain intensity, and analgesic consumption, among patients with migraine in Slovenia.

**Methods:**

This retrospective study analysed 92 adult patients with migraine receiving prophylactic biological treatment at the University Medical Centre Ljubljana. Average number of monthly migraine days (MMD), average analgesic consumption, pain intensity (VAS), and functional disability score (MIDAS) were collected before treatment and after 3 and 12 months. Statistical analysis involved the Friedman test to assess changes over time, followed by Wilcoxon tests with Bonferroni correction for post hoc comparisons.

**Results:**

83 patients completed 12 months of treatment. Significant reductions (p < 0.001) were observed in MMD (median 10 vs. 2 days), analgesic consumption, VAS scores (median 8 vs. 4), and MIDAS scores after 3 months (median 19 vs. 2), sustained at 12 months. Treatment discontinuation occurred in 9 patients due to inefficacy or side effects. Most patients tolerated biologics well, with mild adverse effects reported.

**Conclusions:**

Biological therapies significantly reduced migraine-related symptom burden and functional disability, including decreases in migraine frequency, pain intensity, and analgesic use, in a Slovenian migraine cohort. These findings support the use of biologics as effective and safe options for migraine prevention in real-world clinical practice.

## Izvleček

**Uvod:**

Namen raziskave je bil oceniti učinkovitost biološkega zdravljenja pri zmanjševanju simptomske obremenitve in funkcionalne prizadetosti pri bolnikih z migreno v Sloveniji, vključno z zmanjšanjem mesečnega števila migrenskih dni, intenzivnosti bolečine in porabe analgetikov.

**Metode:**

V retrospektivni študiji smo analizirali 92 odraslih bolnikov z diagnozo migrene, zdravljenih z biološkimi zdravili v Univerzitetnem kliničnem centru Ljubljana. Zbrali smo podatke o povprečnem številu migrenskih dni na mesec (MMD), povprečni porabi analgetikov, intenzivnosti bolečine (VAS) in funkcionalni onesposobljenosti (MIDAS) na treh časovnih točkah: pred začetkom zdravljenja, po 3 in 12 mesecih zdravljenja. Statistična analiza je vključevala Friedmanov test za oceno sprememb skozi čas, sledili pa so Wilcoxonovi testi z Bonferronijevo korekcijo za post hoc primerjave.

**Rezultati:**

Skupno je 83 bolnikov zaključilo 12-mesečno zdravljenje. Po treh mesecih smo zaznali statistično značilno zmanjšanje MMD (mediana z 10 na 2 dni), porabe analgetikov, bolečine po VAS lestvici (mediana z 8 na 4) ter funkcionalne onesposobljenosti po MIDAS lestvici (mediana z 19 na 2), ki so se ohranile tudi po 12 mesecih (p < 0,001). Zdravljenje je v prvem letu prekinilo 9 bolnikov, bodisi zaradi neučinkovitosti ali neželenih stranskih učinkov. Večina bolnikov je biološka zdravila dobro prenašala, pri čemer so bili poročani neželeni učinki večinoma blagi.

**Zaključki:**

Biološka terapija slovenski kohorti bolnikov z migreno pomembno zmanjšuje simptomsko obremenitev in funkcionalno prizadetost, vključno z zmanjšanjem pogostosti napadov, intenzivnosti bolečine in uporabe analgetikov. Ti rezultati podpirajo uporabo bioloških zdravil kot učinkovitih in varnih možnosti za preprečevanje migrene v klinični praksi.

## 1 INTRODUCTION

Migraine is a complex neurological disorder characterised by recurrent attacks of severe headache, typically unilateral and pulsating in nature, accompanied by symptoms such as nausea, vomiting, photophobia, and phonophobia (1).

It affects approximately 14% of the global population (2) and significantly disables patients during their most productive years of life (3). A similar pattern is observed in Slovenia, where migraine substantially impacts patients' daily functioning and contributes to considerable costs related to absenteeism. In 2016, these costs were estimated at €531 per year per patient, highlighting the considerable economic burden of migraine on work productivity (4). Patients with migraine also show a higher prevalence of depression, anxiety and stress disorders, which further worsens their overall health status (5).

Over the past decade, significant progress has been made in the treatment of migraine attacks. Monoclonal antibodies targeting the calcitonin gene-related peptide (CGRP), such as erenumab, galcanezumab, fremanezumab, and eptinezumab, have emerged as effective and well-tolerated options for migraine prevention (6). These prophylactic treatments have demonstrated particular efficacy in reducing the frequency of migraine attacks and alleviating migraine-related symptom burden and functional disability. Erenumab, a monoclonal antibody targeting the CGRP receptor, has been shown in clinical trials to reduce migraine frequency significantly. In patients with chronic migraine, at least a 50% reduction in monthly migraine days was observed in nearly 42% of patients after three months of treatment. Among those with episodic migraine, 57.7% achieved such improvement (7). Additionally, treatment with CGRP monoclonal antibodies has consistently been associated with a reduction in acute medication use and improvements in clinically relevant migraine outcomes. A recent meta-analysis confirmed these benefits across multiple agents, including erenumab, fremanezumab, galcanezumab, and eptinezumab, with significant reductions in monthly migraine days and analgesic use (8).

Despite promising findings in the international literature, there is a lack of clinical data in Slovenia on the impact of biological therapies on migraine-related symptom burden and functional disability. Therefore, this study aims to evaluate their effect on the number of monthly migraine days, pain intensity, migraine-related disability, and the use of analgesics. This study addresses an important evidence gap by providing real-world, longitudinal data from a unique national setting and offering novel insights into the effectiveness of biological therapies for migraine in Slovenia.

## 2 METHODS

### 2.1 Study design and time frame

This study was designed as a quantitative, retrospective analysis aimed at evaluating the impact of biological therapies on migraine-related symptom burden and functional disability. The primary objective was to assess whether treatment with biologics reduces the number of migraine days, analgesic use, headache intensity, and migraine-related functional disability.

### 2.2 Sampling procedure, data collection process, and inclusion criteria

The study included 92 patients aged 18–65 years with a diagnosis of episodic or chronic migraine who received biological therapy at the Outpatient Clinic for Primary Headache Prophylaxis at the Neurology Clinic, University Medical Centre Ljubljana. Only patients with complete clinical measurements at all three assessment points—prior to treatment initiation, at 3 months, and at 12 months—were included in the final analysis. However, patients who discontinued treatment prematurely due to inefficacy or adverse effects (n = 9) were included in analyses of earlier time points where applicable.

Depression was recorded only for patients with a preexisting diagnosis made by specialists (neurologists, psychiatrists, psychologists) prior to their evaluation in our outpatient clinic. Formal psychological testing was not part of routine migraine assessment, and no new diagnoses of depression were made as part of this study. All recorded cases were diagnosed before the initiation of biological therapy for migraine.

### 2.3 Observed outcome

Data were retrospectively collected from existing clinical records in the local information system (Hipokrat, List d.o.o., Ljubljana, Slovenia) and from medical documentation at the outpatient clinic. Monthly data on analgesic consumption and the number of migraine days were additionally obtained from patient-maintained headache diaries. The average number of monthly migraine days (MMD) served as a key indicator of attack frequency, which has direct implications for functionality, productivity, and psychological well-being. A 50% reduction in MMD following biological treatment was interpreted as an improvement in migraine control and a reduced risk of progression to chronic migraine (9). Average monthly analgesic use was used as an additional parameter for estimating treatment effectiveness, with reduced usage also indicating a decreased risk of medication-overuse headache. Migraine-related functional disability and pain intensity were assessed using two standardised instruments: the Migraine Disability Assessment Scale (MIDAS), which evaluates the functional impact of migraine, and the Visual Analogue Scale (VAS), which measures pain intensity on a 0–10 scale. Data were collected at three time points: before treatment initiation, and at 3 and 12 months post-treatment.

### 2.4 Methods of analysis

Data were analysed using IBM SPSS Statistics, version 25. Descriptive statistics were used to summarise the data, including means, medians, and interquartile ranges (IQR), as appropriate. The normality of distribution for continuous variables was assessed using the Shapiro-Wilk test. As the data were not normally distributed, the Friedman test was employed to evaluate differences across the three time points (baseline, 3 months, and 12 months). For post hoc analyses, pairwise comparisons were conducted using the Wilcoxon signed-rank test with Bonferroni correction to account for multiple testing.

### 2.5 Ethical aspects of research

The study was conducted in accordance with ethical guidelines, including the principles of the Declaration of Helsinki. Ethical approval was obtained from the National Medical Ethics Committee of the Republic of Slovenia (approval number: 0120-14/2025-2711-5), and permission to access patient data from the Hipokrat system was granted by the management of the Department of Neurology, University Medical Centre Ljubljana. All patient data were anonymised prior to analysis; therefore, individual informed consent was not required. The study adhered to the ethical standards for data protection and clinical research.

## 3 RESULTS

### 3.1 Sample description

**Figure 1.**
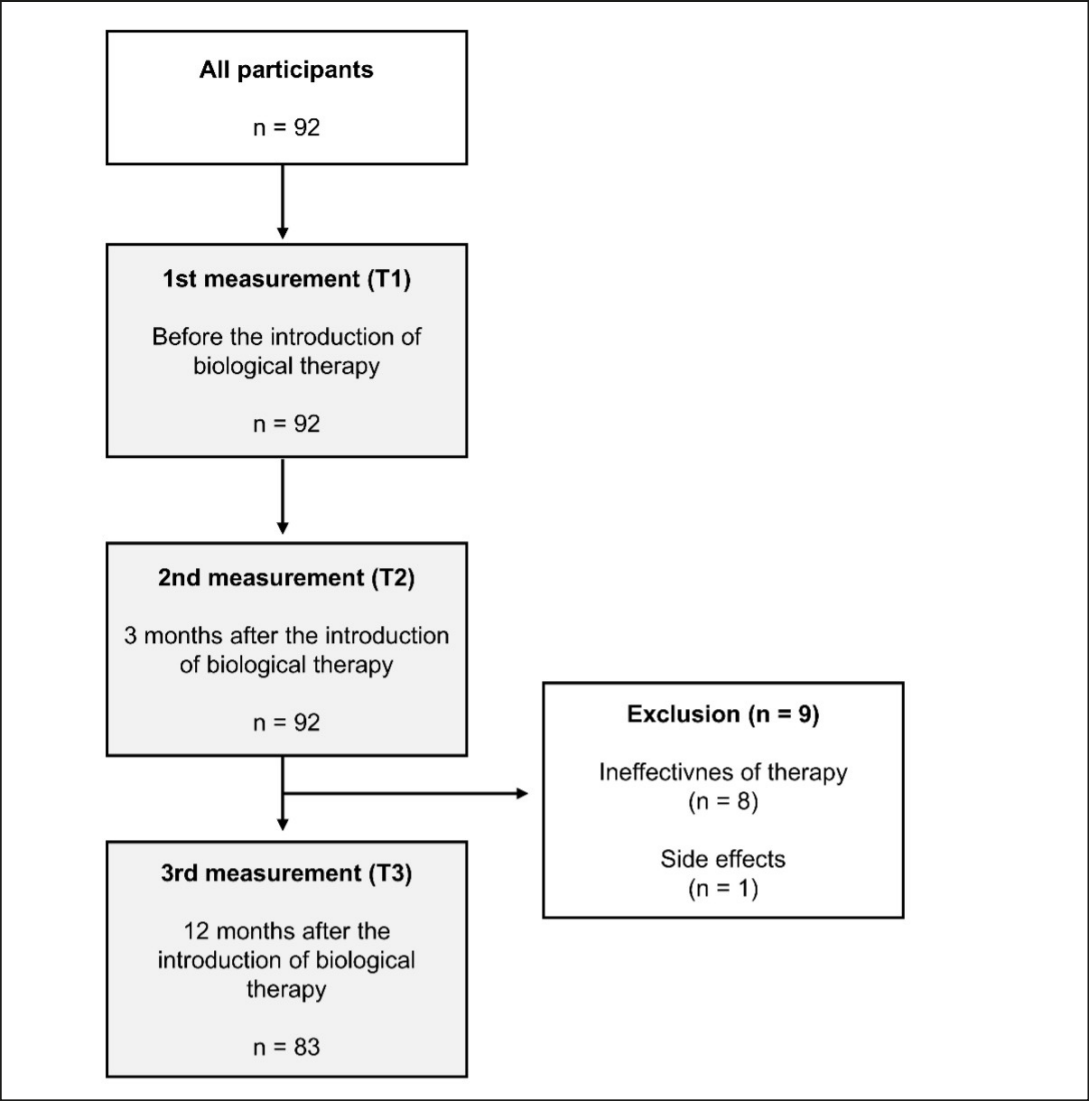
Flowchart of the final patient population.

As shown in Figure 1, the study included 92 patients with migraine who initiated biologic therapy at the Headache Prophylaxis Outpatient Clinic in the Neurology Department in Ljubljana. Of these, 83 patients (90.2%) completed the full 12-month treatment period, while 9 patients (9.8%) discontinued treatment. All patients who discontinued therapy did so after three months of treatment. The primary reason for discontinuation was lack of efficacy (in 8 patients), and one patient discontinued due to adverse effects.

### 3.2 Tables and results overview

Table 1 presents the descriptive statistics of the demographic data and clinical characteristics of the included patients. Table 2 shows the clinical parameter statistics (average MMD, average number of monthly analgesics consumed, VAS, and MIDAS) at three time points (T1, T2, T3), presented as medians with interquartile ranges (IQR).

**Table 1.** Descriptive statistics of the sociodemographic and clinical characteristics of the patients.

**Sociodemographic characteristics of patients**		
**Variable**	**Categories / Units**	**n (%) or mean ± SD**
**Sex**	Male	12 (13%)
	Female	80 (87%)

**Age**		44.5±11.8

**Education**	Primary school	2 (2.2%)
	Secondary school	45 (48,9%)
	College / University	32 (34.8%)
	Master's Degree	4 (4.3%)
	Specialisation	4 (4.3%)
	Doctorate	5 (5.4%)

**Employment**	Yes	71 (77.2%)

**Clinical characteristics of patients**		
**Variable**	**Categories / Units**	**n (%) or mean ± SD**
**Attack frequency**	Episodic migraine	75 (81.5%)
	Chronic migraine	16 (17.4%)

**Duration of migraine (years)**	< 5 years	7 (7.6%)
	5–10 years	23 (25.0%)
	> 10 years	62 (67.4%)

**Type of migraine**	Without aura	78 (84.8%)

**Migraine symptoms**	Nausea	56 (60.9%)
	Photophobia	60 (65.2%)
	Phonophobia	44 (47.8%)
	Osmophobia	8 (8.7%)
	Vomiting	18 (19.6%)
	Fatigue	2 (2.2%)
	No symptoms	7 (7.6%)
	Average number of symptoms	2.0±0.9

**Depression**	Yes	11 (12.0%)

Legend: n = number of patients; SD = standard deviation

**Table 2.** Presentation of the median, IQR, and statistical significance at three time points.

**Variable**	**T1 (median, IQR)**	**T2 (median, IQR)**	**T3 (median, IQR)**	**Statistical significance (p-value)**	
MIDAS	19 [12–30]	4 [0–7]	2 [0–6]	p < 0.001	T1 vs T2: < 0.001^*^
					T1 vs T3: < 0.001^*^
					T2 vs T3: 0.002^*^
VAS	8 [8–9]	4 [3–6]	4 [3–5]	p < 0.001	T1 vs T2: < 0.001^*^
					T1 vs T3: < 0.001^*^
					T2 vs T3: 0.351
Average analgesic consumption	12.5 [9–19.5]	4 [1–6]	2 [2–4]	p < 0.001	T1 vs T2: < 0.001^*^
					T1 vs T3: < 0.001^*^
					T2 vs T3: 0.009^*^
Average number of monthly migraine days (MMD)	10 [7–12.5]	3 [2–5]	2 [1.5–3]	p < 0.001	T1 vs T2: < 0.001^*^
					T1 vs T3: < 0.001^*^
					T2 vs T3: 0.024

Legend: MMD = monthly migraine days; MIDAS = Migraine Disability Assessment questionnaire; VAS = Visual Analogue Scale for pain intensity;

* = statistically significant after Bonferroni correction, α = 0.017

Following the initiation of biological therapy, there was a statistically significant reduction in VAS, MMD, average analgesic consumption, and MIDAS (p < 0.001). The most significant improvement was observed after three months of treatment, with results sustained at one-year follow-up. However, after applying the Bonferroni correction (p = 0.017), statistically significant differences between T2 and T3 were found for MIDAS (p = 0.002) and analgesic use (p = 0.009), while no statistically significant differences were observed for the other two variables.

These findings confirm the clinically meaningful effects of treatment, as patients reported a decrease in MMD, less intense migraine attacks, reduced need for acute therapy, and decreased migraine-related functional disability. At the one-year mark, 83 of the 92 included patients continued treatment; 9 discontinued due to therapy inefficacy or side effects.

**Figure 2.**
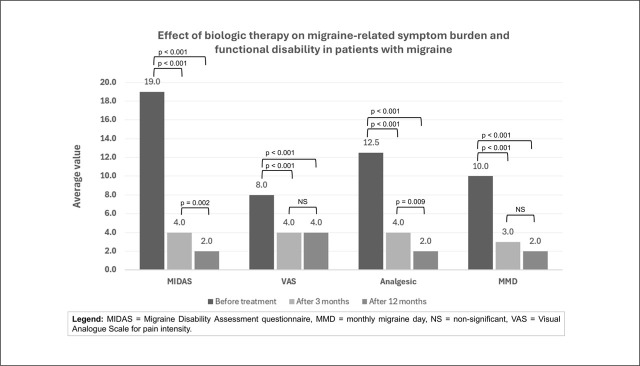
Effect of biological therapy on migraine-related symptom burden and functional disability in patients with migraine.

The results are summarised in Figure 2, which illustrates a statistically significant decrease across all measured values, indicating an overall improvement in migraine-related symptom burden and functional disability.

Table 3 shows the current prophylaxis with biological therapies and reported adverse effects, providing insight into the reasons for therapy initiation and an assessment of its safety.

**Table 3.** Overview of current biological prophylaxis and reported side effects.

		**n (%)**
**Current prophylaxis with biological therapy**	Erenumab (Aimovig)	13 (14.1%)
	Fremanezumab (Ajovy)	45 (48.9%)
	Galcanezumab (Emgality)	26 (28.3%)
	Eptinezumab (Vyepti)	8 (8.7%)

**Side effects of biological therapy**	Pain at injection site	3 (3.3%)
	Leg cramps	2 (2.2%)
	Dizziness	2 (2.2%)
	Itching	4 (4.3%)
	Rash- allergic reaction	1 (1.1%)
	Redness	7 (7.6%)
	Swelling	2 (2.2%)
	Runny nose	1 (1.1%)
	Constipation	2 (2.2%)
	No side effects	72 (78.3%)

In addition to the group of patients monitored at three time points over a one-year period, we also analysed patients who discontinued treatment prematurely. Table 4 presents data on 9 patients who stopped biological therapy, representing 9.8% of all included participants (92 patients). The analysis includes the demographic and clinical characteristics of the treatment in these patients.

**Table 4.** Overview of characteristics of patients who discontinued treatment.

**Characteristics**	**N = 9**	**%**
**Female**	7	77.8%
**Type of migraine (chronic migraine)**	5	55.6%
**Employed**	5	55.6%
**Duration of migraine in years (>10 years)**	6	66.7%
**Reason for discontinuation:**		
• Ineffectiveness of treatment	8	88.9%
• Side effects	1	11.1%
**Tension-type headache**	4	44.4%
**Comorbid depression**	4	44.4%

## 4 DISCUSSION

Results of the study confirm that treatment of migraine with biological therapies significantly and durably reduces the frequency of migraine attacks, pain intensity, and analgesic consumption, thereby markedly improving patients' daily functioning. The effects were evident after three months of treatment and were maintained or further improved after twelve months, consistent with previous studies (10, 11).

The treatment was investigated in a cohort of 92 patients, of whom 87% were female, aligning with epidemiological data indicating that migraine is three times more common in women (9). The mean age of 44.5 years reflects a population in the active phase of life, particularly susceptible to the impacts of migraine on daily functioning and work productivity. The high employment rate (77%) further underscores the importance of effective therapy to reduce absenteeism and improve productivity, both of which are crucial from a public health perspective.

Most patients had episodic migraine (81.5%), which is consistent with the epidemiological prevalence of this subtype in the general population. However, this distribution might seem unexpected in a tertiary care setting, where a higher proportion of patients with high-frequency episodic or chronic migraine would typically be anticipated. Treatment effects were comparable across episodic and chronic migraine, demonstrating the broad efficacy of biological therapies regardless of migraine type. The presence of migraine for more than 10 years in the majority of patients (67.4%) indicates prolonged disease burden and highlights the need for targeted and effective therapies. Although migraine is a chronic condition, the therapeutic response to biological treatments was pronounced and sustained, consistent with findings in the literature that early and consistent treatment contributes to better outcomes.

The study also confirmed a favourable safety profile of biological therapies — 79.3% of patients reported no side effects, with the most common adverse effects being mild local reactions at the injection site, consistent with existing clinical trial data (11, 12). Additionally, only one of 9 patients who failed to complete one year of treatment has discontinued treatment due to side effects. High treatment tolerability is important for good therapeutic relationships and treatment adherence. Interestingly, despite being a common side effect in similar studies, the incidence of constipation in our patient cohort was relatively low.

The use of standardised measurement instruments such as MIDAS and VAS enabled quantitative monitoring of functional improvements and pain reduction. Results showed statistically significant improvements as early as the first three months, and the effect was sustained after one year of treatment, underscoring the importance of assessing long-term efficacy.

Despite positive outcomes, it is important to note that 9.8% of patients discontinued treatment prematurely, mainly due to ineffectiveness. These patients often had a longer disease history and greater comorbid psychological burdens, such as depression. The association between depression and poor treatment response is well documented in the literature and warrants additional attention in the management of migraine patients (13).

The study is limited by a retrospective design, a small sample size, and the lack of a control group, which may limit the generalisability of the results and warrant cautious interpretation. Nevertheless, the results offer valuable real-world data on the efficacy and safety of biological therapies in clinical practice.

It is also important to highlight the key role of nurses in patient monitoring, education, and support during treatment. Through a comprehensive approach that includes regular monitoring of clinical parameters and patient support, nurses significantly contribute to increased adherence and, in turn, more successful treatment outcomes.

## 5 CONCLUSIONS

The results of this study confirm the significant clinical impact of biological therapies in the treatment of migraine. Using real-world, longitudinal data from a national clinical setting, this study provides novel evidence on the effectiveness of biological therapies for migraine in routine practice. A statistically significant reduction in the number of migraine days, analgesic use, pain intensity, and functional impairment indicates both a rapid and sustained improvement in patients' condition already within three months of treatment, with a continued trend of improvement over the course of one year.

Biological therapies are effective and well-tolerated treatment options for patients with moderate to severe migraine who do not respond to standard preventive treatments, leading to a substantial reduction in migraine-related symptom burden and functional disability. The favourable safety profile further supports their suitability for long-term use.

Based on our findings, we emphasise the importance of an individualised treatment approach, regular monitoring of therapeutic response, and a multidisciplinary model of care, in which the nurse plays a key role in patient education and support. These results demonstrate that biological therapies represent a major advancement in modern migraine management, contributing meaningfully to improving patients' daily functioning.
